# Serotonin, glutamate and glycerol are released after the injection of hypertonic saline into human masseter muscles – a microdialysis study

**DOI:** 10.1186/1129-2377-15-89

**Published:** 2014-12-17

**Authors:** Sofia Louca, Nikolaos Christidis, Bijar Ghafouri, Björn Gerdle, Peter Svensson, Thomas List, Malin Ernberg

**Affiliations:** 1 Orofacial Pain and Jaw Function, Department of Dental Medicine, Karolinska Institutet, Huddinge, Sweden; 2 Scandinavian Center for Orofacial Neuroscience (SCON), Stockholm, Sweden; 3 Department of Pain and Rehabilitation Center and Department of Medical and Health Sciences, Linköping University, SE-581 85 Linköping, Sweden; 4 Section of Clinical Oral Physiology, School of Dentistry, University of Aarhus, Vennelyst Boulevard 9, DK-8000 Aarhus C, Denmark; 5 Department of Stomatognathic Physiology, Faculty of Odontology, Malmö University, Malmö, Sweden

**Keywords:** Hypertonic saline, Pain, Masseter muscle, Biomarkers

## Abstract

**Background:**

Chronic myalgia is associated with higher muscle levels of certain algesic biomarkers. The aim of this study was to investigate if hypertonic saline-induced jaw myalgia also leads to release of such biomarkers and if there were any sex differences in this respect.

**Methods:**

Healthy participants, 15 men and 15 aged-matched women (25.7 ± 4.3 years) participated. Intramuscular microdialysis into masseter muscles was performed to sample serotonin (5-HT), glutamate, lactate, pyruvate, glucose and glycerol. After 2 hours 0.2 mL hypertonic saline (58.5 mg/mL) was injected into the masseter on one side and 0.2 mL isotonic saline (9 mg/mL) into the contralateral masseter close to the microdialysis catheter. Microdialysis continued for 1 hour after the injections. Pressure pain thresholds (PPT) and pain were assessed before and after injections.

**Results:**

The median (IQR) peak pain intensity (0–100 visual analogue scale) after hypertonic saline was 52.5 (38.0) and after isotonic saline 7.5 (24.0) (p < 0.05). 5-HT, glutamate and glycerol increased after hypertonic saline injection (p < 0.05). Lactate, pyruvate and glucose showed no change. PPT after microdialysis was reduced on both sides (p < 0.05) but without side differences. Pain after hypertonic saline injection correlated positively to 5-HT (p < 0.05) and negatively to glycerol (p < 0.05).

**Conclusions:**

5-HT, glutamate and glycerol increased after a painful hypertonic saline injection into the masseter muscle, but without sex differences. Since increased levels of 5-HT and glutamate have been reported in chronic myalgia, this strengthens the validity of the pain model. Glycerol warrants further investigations.

## Background

Chronic temporomandibular disorder (TMD) pain has a prevalence of approximately 10–15% in the adult population [1,2]. Myofascial TMD is the most frequent type of TMD pain in the orofacial region and is often accompanied by restricted mouth opening, pain upon chewing, muscle soreness, pain referral and headache which reduces patients’ quality of life [3,4]. The prevalence of TMD is known to be 1.5 to 2 times higher amongst women than men [5,6]. However, the etiology of myofascial TMD and its higher prevalence in women is still not fully understood.

Stress- related muscular hyperactivity, such as tooth clenching/grinding, is often suggested to contribute to the etiology of myofascial TMD [7,8] although no simple relationship between electromyographic (EMG) activity and TMD pain has been noted [9,10]. Epidemiological studies also showed a greater likelihood of myofascial pain in the presence of self-reported tooth clenching [11,12]. Furthermore, it has been suggested that muscular hyperactivity could cause pain by mechanical overloading accompanied by disturbed local blood flow [13].

As a result, this may lead to ischemia and release of inflammatory, as well as, neuroactive biomarkers, such as neuropeptides, bradykinin, protons, serotonin (5-HT), glutamate and cytokines. When these inflammatory and neuroactive biomarkers activate receptors on peripheral sensory afferents, for instance 5-HT_3_, N-methyl-D-aspartic acid (NMDA), 2-amino-3-(3-hydroxy-5-methyl-isoxazol-4-yl) propanoic acid (AMPA) and kainate receptors, they induce pain [14,8].

Several studies have shown that patients with chronic myalgia have higher interstitial levels of 5-HT and glutamate in the masseter and trapezius muscles compared to healthy controls, and that muscle levels correlated to pain levels [15-20]. Intramuscular injection of serotonin and glutamate further evoked pain [21,22]. Thus these substances can be regarded as potential biomarkers for chronic myalgia.

In addition, interstitial levels of lactate and pyruvate increased in painful trapezius muscle, which may also indicate a role for metabolic markers in chronic myalgia [17,18,23,24]. Only one previous study has studied the release of lactate and pyruvate in myofascial TMD pain and found similar levels compared to pain-free controls [25].

To better understand and widen the knowledge about chronic pain, use of experimental pain models that mimic pain conditions are necessary. Intramuscular injections of hypertonic saline are frequently used to mimic human myalgia and regarded as a valid model of myalgia, in the orofacial region [26]. This experimental pain model has an acute character and causes a short lasting, distinct sensation of deep, diffuse pain, and pain referral [27-30]. However, to increase validity of the experimental pain model, the same biomarkers as in clinical pain should be increased after hypertonic saline injections. Therefore the main aim of this study was to investigate if jaw muscle pain induced by hypertonic saline lead to a release of muscle biomarkers. A second aim was to investigate if there were any sex differences related to the release of mediators. We hypothesized that release of several inflammatory mediators, such as 5-HT, glutamate and other biomarkers of metabolic activity, such as lactate, pyruvate, glucose and glycerol were significantly higher in hypertonic saline-induced pain and that there was higher release in women than in men.

## Methods

### Participants

Healthy participants of both sexes participated in the study. The participants were recruited by advertisements but additionally from colleagues and students at the Department of Dental Medicine at the Karolinska Institutet, Sweden, where the study was conducted. No students from current courses given by the authors were included. Inclusion criteria were age over 18 years and good general health. Exclusion criteria were any current orofacial pain, diagnosed systemic muscular or joint diseases, such as fibromyalgia, rheumatoid arthritis, whiplash-associated disorder, neuropathic pain or neurological disorders, pregnancy or lactation, high blood pressure and use of antidepressants or analgesics during the last three days.

According to the power calculation, inclusion of 26 participants would be sufficient to detect a statistically significant difference of 20% (SD 30%) in biomarker level between interventions at a significance level of 5% with a power of 90%. In order to compensate for dropouts/missing data four additional participants were included.

The project followed the guidelines according to the Declaration of Helsinki as well as Good Clinical Practice and was approved by the regional ethical review board in Stockholm (2008/362-31) and the local radiology committee (Dnr 11/08) at Karolinska University Hospital in Huddinge, Sweden. All participants received written and verbal information about the study before participating and gave their written consent.

### Experimental protocol

This study used a randomized, double-blinded and placebo-controlled design in which hypertonic saline was injected into one of the masseter muscles and isotonic saline (control) on the contralateral side. Randomization of sides for injection was performed by a computer (http://www.randomization.com). The preparation and blinding of syringes was made by a researcher not participating in the study and the syringes were marked with the participants study number. Since hypertonic saline and isotonic saline have identical appearance, both the participant and the investigator were blinded.

**Figure 1:**
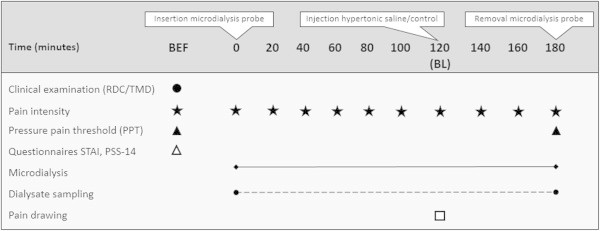
**Flow chart of the experimental setting.** The figure shows the time points (minutes) for the clinical examination, assessment of pain intensity, pressure pain threshold, questionnaires (STAI, PSS-14, MPQ) including pain drawing, microdialysis and dialysate sampling in 15 healthy men and 15 age-matched women. BEF = Before microdialysis; BL = Baseline at 120 minutes; RDC/TMD = Research diagnostic criteria; STAI = State-Trait Anxiety Inventory; PSS = Perceived Stress Scale.

In order to confirm that the participants were eligible for inclusion in the study screening was performed including a clinical examination according to the Research Diagnostic Criteria for TMD (RDC/TMD) Axis I [31]. After inclusion in the study the participants filled in instruments regarding anxiety including the State-Trait Anxiety Inventory (STAI-trait) [32] and stress by the Perceived Stress Scale (PSS) [33]. Further, the pressure pain threshold (PPT) was assessed bilaterally over the masseter muscles and over the right forefinger.Participants were placed in a conventional dental chair and were instructed to lie as still as possible during the experiment and to avoid talking. After baseline registrations and local anesthesia bilateral microdialysis was performed over 3 hours. The participants received a hypertonic saline injection into one of masseter muscle and an isotonic saline injection on the contralateral side, and pain intensity, pain duration and pain area evoked by the injections were registered. After microdialysis, PPT was assessed again at the same sites (Figure 1).

### Assessments of pain and pressure pain threshold

A 0–10 numeric rating scale (NRS) with the end-points marked “no pain” and “the worst pain experienced” was used to assess pain in the masseter muscle before the start of the study to ensure that all participants were pain free, in that they graded their pain as 0, and thereafter every 20th minute during microdialysis when the dialysate microvials were changed.

The PPT was assessed with an electronic algometer (Somedic Sales AB, Höör, Sweden) with a standardized pressure rate of 50 kPa per second [15,34]. The tip of the algometer was 1 cm^2^ and covered with a 1-mm thick rubber pad. Pressure was administrated on the most prominent point of the masseter muscle, which was verified during contraction and the PPT was assessed in the relaxed muscle. The participants were instructed to press the signal button immediately when the sensation of pressure turned into pain. The right forefinger was used as a reference. All recordings were performed three times with an interval of 2 minutes between measurements. The average of the three recordings was used in statistical analyzes.

### State-Trait Anxiety Inventory (STAI)

The STAI is an instrument used to measure anxiety in adults. It comprises two twenty-item scales that determine two types of anxiety [32,35]. The first measures state anxiety, which is anxiety about an event, and the second measures trait anxiety, which is anxiety level as a personal characteristic. Questions have a range of four possible responses, rated on a scale from 1–4. Several of the items had reversed scoring. In this study the focus was on the more general and long-standing quality of trait anxiety (STAI-trait), which is very common characteristic in patients with chronic pain [32,35]. Scores ranged from 20 to 80, with higher scores indicating higher levels of anxiety. If the scores were ≤ 30 the patient showed no or low signs of anxiety but if the scores were > 30, patients showed moderate to high signs of anxiety.

### Perceived stress scale-14 (PSS-14)

The PSS questionnaire is a widely and commonly used questionnaire of psychological nature that measures the perception of stress. It consists of 14 stress-related questions of a general nature, including questions regarding feelings and thoughts during the last month, and also what situations in life that are perceived as stressful and the current levels of experienced stress. The questionnaire was used according to Cohen and co-workers [33] by reversing the scores on the seven positive items and then summing the values of the 14 questions. The PSS questionnaire had a total sum of 56. A score below 23.67 has been considered as normal in healthy participants [33,36].

### Microdialysis

Intramuscular microdialysis in the masseter muscle was performed to sample 5-HT, glutamate, lactate, pyruvate, glucose and glycerol, and to estimate nutritive muscle blood flow. The most prominent point of the masseter muscle was chosen and verified by palpation as the participants were instructed to clench. To anaesthetize the skin overlying the muscle a local anaesthetic patch (EMLA® patch, lidocaine/prilocaine 25 mg/25 mg, AstraZeneca AB, Södertälje, Sweden) was applied for 30 minutes. The EMLA® patch was then removed and the skin surface thoroughly cleaned with injection swabs (70% isopropyl alcohol). A sterile 6-mm thick plastic plate (10 × 40 mm) was placed over the chosen point of the masseter muscles and fixed with surgical tape. The plate contained two 1.3-mm wide canals, 10 mm apart, one at 90° angle to the surface and the other at 45° angle. A standard catheter (Ø 1.3 × 32 mm, Venflon, BOC Ohmeda AB, Helsingborg, Sweden) was inserted intramuscularly parallel to the muscle fibers to a depth of approximately 20 mm from the skin surface, guided by the 45° canal of the plastic plate. When inserting the catheter a slight resistance was typically felt upon penetration of the muscle fascia, after it was inserted 12 mm into the muscle to create a canal in the muscle, for use during insertion of the microdialysis probe (see below). The catheter was then withdrawn 10 mm, the needle removed and the external part of the catheter cut with a scalpel at its entrance into the plastic plate. In this manner the tip of the plastic tube was placed approximately 10 mm deep into the muscle [37].

A sterile flexible microdialysis probe (Ø 0.5 mm; membrane length 10 mm, total length 30 mm; molecular cut off: 6 kDa MAB 11, Microbiotech AB, Stockholm, Sweden) was inserted into the muscle via the catheter to its full length to ensure that the whole membrane protruded outside the catheter. The probe was perfused (5 μl/min) with a Ringer-Acetate solution containing 0.5 mM Ringer-lactate with a 2-ml syringe connected to a micro infusion pump (MAB 140, Microbiotech AB, Stockholm, Sweden). A volume of 3.0 μL [^14^C]-lactate (specific activity: 7.4 MBq/mL; PerkinElmer Life Sciences, Boston, MA, USA) was added to the perfusion medium to determine the relative recovery (RR) of the mediators [38] and 3.0 μL ^3^H_2_O to estimate blood flow [19,39]. Samples of 120 μL were collected every 20 minutes in 200-mL capped microvials. The samples were immediately frozen (−70°C).

The whole microdialysis experiment lasted for 3 hours. After 2 hours of stabilization (trauma phase), hypertonic or isotonic saline was injected into the masseter. Microdialysis continued for 1 hour after the injections. When the microdialysis was finished the probe and the plastics were removed as a unit and the membrane of the probe was checked to make sure that no damaged occurred to it and that the whole membrane protruded outside the catheter.

### Injections

Two standard disposable 19 mm needles (Neofly, BOC Ohmeda AB,) with a diameter of 0.4 mm were used for injection of test substances and inserted to their full length into each of the masseter muscles guided by the 90° canal of the plastic plate. The needles were inserted into the muscles directly after probe insertion to prevent any pain caused by the needle that would interfere with any pain from the injections. By using the guide, the needle and the probe were in close proximity in the muscle, as described previously [37].

Simultaneous bilateral injections of 0.2 mL sterile hypertonic saline (58.5 mg/mL NaCl, Karolinska University Hospital Pharmacy) into the masseter muscle on one side and 0.2 mL isotonic saline (NaCl 9 mg/mL, Fresenius Kabi, Uppsala, Sweden) into the contralateral masseter muscle was assured with the aid of an infusion pump (Harvard Infusion Pump 22, Harvard Apparatus, Edenbridge, UK). The substances were injected over 10 seconds using an infusion rate of 1200 μL/min.

A 100-mm visual analogue scale (VAS) with the end-points 0 = no pain and 100 = the worst pain experienced was used to evaluate pain intensity in the masseter muscles after injections. This was done every 15th second until pain had subsided, to a maximum time of 300 seconds [34]. From these measurements, individual maximum pain intensity (VAS peak) and pain duration were calculated. Pain duration was defined as the pain assessed by the participants immediately after injections until it had faded away, according to a previous study [34]. Pain drawings of the lateral side of the head (each side separately) were also used in order to register the pain area (au) including the maximum pain distribution after the injections.

### Analyzes

The first 2 hours of the microdialysis were regarded as a trauma phase and no dialysate samples were analyzed. The microdialysate samples were analyzed at the Painomics Laboratory, Rehabilitation Medicine, Department of Medical and Health Sciences, Linköping University, Sweden. The concentrations of 5-HT were analyzed with high-pressure liquid chromatography, with electrochemical detection as previously has been described [18]. The detection limit was 20 fmol/10 μL.

Other biomarkers (glutamate, glucose, lactate, pyruvate and glycerol), were analyzed with the ISCUS® analyzer (ISCUS, Dipylon Medical AB, Solna, Sweden). The limits of detection (LOD) were 1.0 μmol/L for glutamate, 0.1 mmol/L for glucose and lactate, 10 μmol/L for pyruvate and 0.22 mg/mL for glycerol. Samples with concentrations below 50% of LOD were reported as having the same concentration as LOD, whilst samples with concentrations above 50% of LOD, the concentration obtained was reported.

To determine the RR, 5 μL of each dialysate and perfusate was pipetted into a counting vial containing 3 mL scintillation fluid (High-flash Point, Universal LSC-Cocktail, ULTIMA GOLD™, PerkinElmer, Inc.) and vortexed. The counts per minutes (cpm) for [^14^C]-lactate and ^3^H_2_0 were analyzed in a liquid scintillation beta counter (Beckman LS 6000TA; Beckman Instruments, Inc., Fullerton, CA, USA). RR was calculated as (cpm_p_-cpm_d_)/cpm_p_, where cpm_p_ and cpm_d_ were cpm in the perfusate and dialysate, respectively. The RR calculation describes the ratio between the actual concentration in the muscle tissue and the concentration of the dialysates. Interstitial concentrations (C_i_) were calculated for glutamate, glucose, lactate, pyruvate and glycerol as C_i_ = [C_d_ - C_p_]/ RR + C_p_, where C_d_ was the dialysate concentration and C_p_ was the perfusate concentration [33,38]. Nutritive blood flow was calculated using ^3^H_2_O with the formula 1/(cpm_d_/cpm_p_), where cpm_d_ was ^3^H_2_O counts per min in the dialysate and cpm_p_, in the perfusate [19,39]. RR and blood flow were analyzed at all time-points for the entire microdialysis.

### Statistics

Data were analyzed with SigmaPlot for Windows, version 11 (Systat Software Inc., Chicago, IL, USA) and SPSS software version 15.0 (SPSS Inc. Chicago, IL, USA). Non-parametric statistics were used to analyze the substances since they were not normally distributed and attempts to transform data did not change this. For descriptive statistics median and interquartile range (IQR) were used. Friedman test was used to analyze changes in substances and pain levels over time at each side separately. Bonferroni correction was used as post-hoc test when the Friedman test indicated a significant difference. To analyze differences between hypertonic saline and control sides at the different time points, the Mann–Whitney *U*-test was used. In addition the maximum pain intensity (VAS peak) induced by the injections was compared between sides with Mann–Whitney *U*-test.

Differences in PPTs between sides before and after microdialysis and differences between sexes were analyzed with unpaired t-test. The Mann Whitney *U*-test was used to test the differences in VAS peak and pain area between sides and differences in these variables between sexes. Also differences between sexes in STAI-trait and PSS-14 scores were tested with the Mann Whitney *U*-test.

Spearman’s correlation test, adjusted for multiple testing with Bonferroni correction, was used for analyzes of significant correlations between NRS pain and release of biomarkers at 140 minutes. For all tests the level of significance was set to p < 0.05.

## Results

Thirty healthy participants, 15 men and 15 aged- matched women, with a mean age of 25.7 (SD 4.3) years participated in the study. None of the subjects dropped out.

The PSS and STAI-trait questionnaires indicated stress and anxiety levels within the normal range in both the men and women (Table 1).

**Table 1:** The median (IQR) Perceived stress (PSS) and Trait Anxiety (STAI-trait) at baseline

	**PSS**	**STAI-trait**
All	15.5 (10.0)	31.0 (6.8)
Men (n = 15)	15.0 (8.3)	31.5 (5.0)
Women (n = 15)	16.0 (10.8)	30.0 (8.8)

IQR = interquartile range (75 percentile minus 25 percentile).

There were no significant differences between sexes.

### Pain evoked by insertion of the microdialysis probe

No participant reported any pain before microdialysis. The catheter and microdialysis probe induced a small degree of pain on both sides in 10 out of 30 participants. Twenty minutes after probe insertion the median (IQR) pain for those participants was NRS 1 (1.0). The pain levels during the trauma phase, for each participants were 0 (0) on both sides at all time points, except of hypertonic saline at 20 min, where it was 0 (0.8) and on the control side at 20 min as well as 40 min where it was 0 (1.0). No significant differences between sides were found at any time points during the stabilization period (before injections).

### Pain characteristics after injections

**Figure 2:**
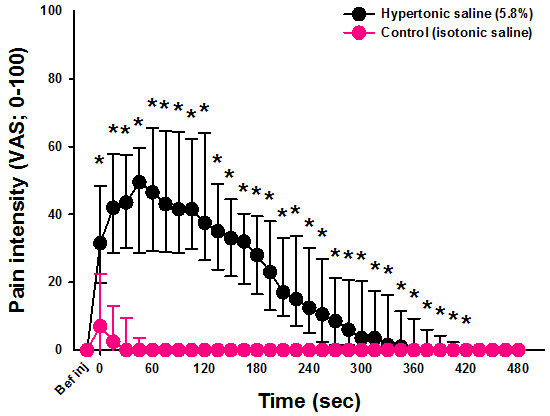
**Pain intensity on the hypertonic saline side and control side.** Graph showing the median (IQR) pain intensity according to VAS at the hypertonic saline and the control sides throughout the experiment. Before injection there were no differences between sides. After injections, the pain intensity was significantly higher and the pain duration was significantly longer on the side injected with hypertonic saline (Mann Whitney *U*-test; p < 0.05).

**Figure 3:**
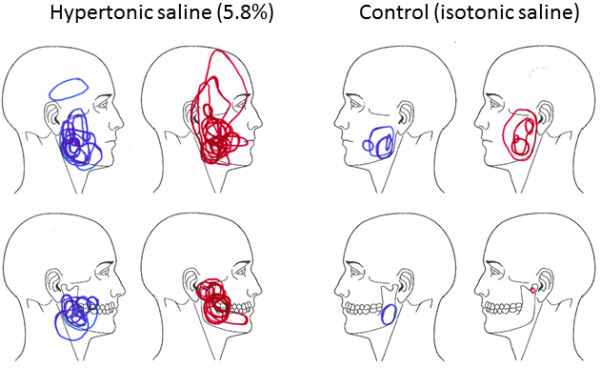
**Pain distribution during experimental pain in the masseter muscle.** Figures showing the pain distribution in the masseter muscle after bilateral injections of hypertonic saline on one side and isotonic saline on the contralateral side in 30 healthy participants (15 women and 15 age-matched men). The red areas show the pain distribution in women and the blue areas indicate the pain spread in men. The pain area was significantly larger after injection of hypertonic saline than control (Mann Whitney *U*-test; p < 0.05).

Hypertonic saline injection evoked pain of moderate intensity (p < 0.001) that lasted for more than 5 min, whereas isotonic saline induced only minor and very short-lasting pain (p < 0.001). The post-hoc test showed that the intensity was significantly higher at the hypertonic saline side 0–420 seconds after injections (Figure 2). The VAS peak was significantly higher, the pain duration significantly longer and the pain area significantly larger on the side injected with hypertonic saline compared to the control side (Table 2 and Figure 3).

**Table 2:** The median (IQR) peak pain (VAS), pain duration (s) and pain area (au) after injections

	**Hypertonic saline side**	**Control side**
Peak pain		
All	52.5 (38.0)	7.5 (24.0)*
Men	50.0 (30.3)	0.0 (24.3)*
Women	56.0 (50.5)	15.0 (22.0)*
Pain duration		
All	345 (150)	8 (45)*
Men	405 (170)	0 (64)*
Women	315 (131)	15 (45)*
Pain area		
All	54 (65.0)	0 (3.0)*
Men	46 (69.3)	0 (2.3)*
Women	60 (87.8)	0 (5.5)*

Table shows data from thirty healthy participants, 15 men and 15 aged-matched women.

IQR = interquartile range (75 percentile minus 25 percentile) VAS = 100 mm visual analogue scale * = significant difference between sides (*p* < 0.001). There were no significant differences between sexes.

### Pressure pain thresholds

The PPTs before and after microdialysis are shown in Table 3. There were no significant differences in baseline PPT values between masseter muscle sides. After completion of the microdialysis the PPT on the hypertonic saline side decreased by 18% and on the control side by 27% compared to baseline. No significant difference was found over the reference point (finger) before and after microdialysis.

**Table 3:** The mean (SD) pressure pain threshold (PPT; kPa) of the masseter muscles before and after microdialysis

	**Hypertonic saline side**	**Control side**	**p-values between sides**	**Reference point**
Before				
All	283.1 (82.6)	299.0 (185.0)	0.619	747.4 (217.8)
Men	318.0 (70.5)¤	291.2 (70.2)	0.306	836.1 (193.7)
Women	245.6 (80.1)	307.4 (261.3)	0.308	658.7 (209.4)
After				
All	232.1 (83.8)*	217.5 (85.5)*	0.407	742.0 (193.2)
Men	250.1 (83.4)*	234.6 (96.3)	0.642	766.8 (194.6)
Women	214.1 (83.0)	200.4 (72.4)	0.634	717.1 (195.2)

Data from thirty healthy participants, 15 men and 15 aged-matched women.

* = significantly lower PPT after microdialysis (p < 0.05).

¤ = significantly higher PPT compared to women (p < 0.05).

### Release of biomarkers, relative recovery and blood flow

**Figure 4:**
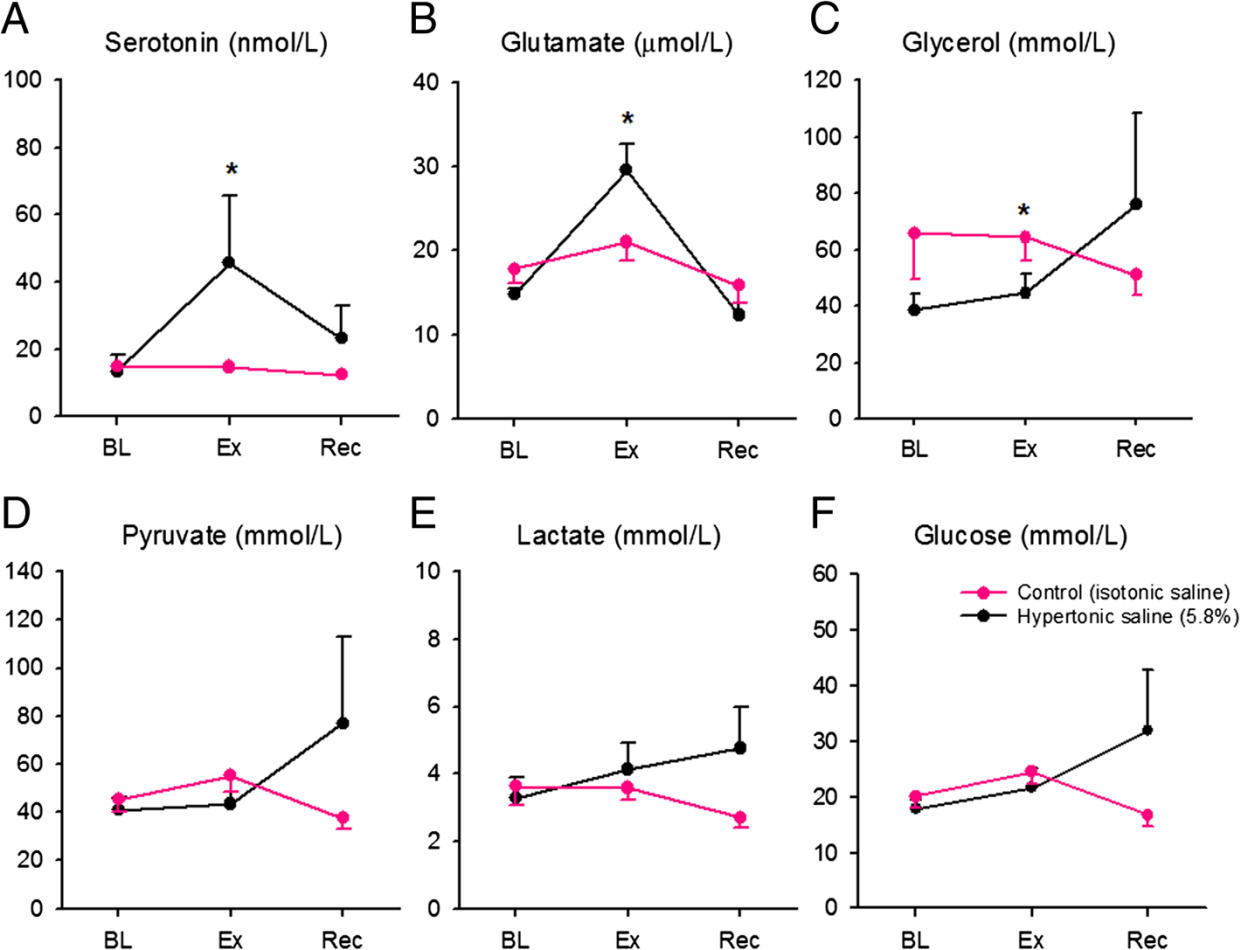
**Release of biomarkers in the masseter muscle during experimental pain.** Graph shows the release of serotonin **(A)**, glutamate **(B)**, glycerol **(C)**, puruvate **(D)**, lactate **(E)** and glucose **(F)** during the baseline (BL), experimental (Ex) and recovery (Rec) phases of the microdialysis. There was a significant increase of serotonin and glutamate on the hypertonic saline side over time (Friedman test; p < 0.05) and at time point 140 minutes compared to baseline (Bonferroni post-hoc test; p < 0.05), but no change on the control side. Glycerol increased significantly at the time point 140 minutes (Bonferroni post-hoc test; p < 0.05) on the hypertonic saline side but not the control side. The other substances; lactate, pyruvate and glucose show no significant increase.

The levels of mediators are shown in Figure 4. The level of 5-HT changed significantly over time (p < 0.05) on the hypertonic saline side. The post-hoc test showed a significant increase at 140 minutes, corresponding to the first dialysate obtained after injection of hypertonic saline, compared to baseline (Figure 4). There were no changes in the 5-HT levels on the control side at any time-point (Figure 4A).

There was a significant difference in glutamate over time (p < 0.05; Friedman test) on the hypertonic saline side. The post-hoc test showed a significant increase at 140 minutes compared to baseline. There were no significant time differences on the control side (Figure 4B). Glycerol changed significantly over time (p < 0.05; Friedman test) and was increased at the time point 140 minutes (p < 0.05) on the hypertonic saline side. However, no changes were found on the control side (Figure 4C). None of the mediators lactate, pyruvate and glucose, differed significantly over time or between sides (Figures 4D-3F). Neither were there any significant differences in any of the mediators on the control side. The mean RR and blood flow are shown in Table 4. The mean RR varied between 22 (14)% and 31 (9)% on the hypertonic saline side, and between 19 (10)% and 37 (12)% on the control side. Blood flow did not differ between sides or change as a consequence of injections.

**Table 4:** The mean (SD) relative recovery (%) and blood flow (%) in the masseter muscle during microdialysis

**Minutes**	**Hypertonic saline side**		**Control side**	
	**Relative recovery**	**Blood flow**	**Relative recovery**	**Blood flow**
20	31 (9)	43 (19)	37 (12)	41 (22)
40	27 (9)	43 (18)	29 (14)	42(22)
60	28 (9)	42 (19)	28 (10)	41 (21)
80	28 (10)	42 (21)	25 (14)	42 (21)
100	29 (11)	36 (23)	29 (9)	43 (19)
120	23 (10)	39 (19)	21 (9)	43 (16)
140	22 (14)	41(21)	19 (10)	38 (21)
160	23 (9)	40 (19)	23 (8)	41 (20)
180	24 (9)	42 (21)	20 (10)	42 (19)

There were no significant differences in relative recovery or blood flow at any time-point.

### Correlations between pain and biomarker levels

The NRS pain intensity at 140 minutes on the hypertonic saline side correlated positively to the 5-HT level (r_s_ = 0.408; n = 27, p = 0.034) and negatively to the glycerol level (r_s_ = −0.399; n = 28, p = 0.035) in the first dialysate sampled after injection (120–140 minutes). There were also significant negative correlations on the control side between NRS pain intensity and glutamate (r_s_ = −0.551; n =28, p = 0.003), glycerol (r_s_ = −0.407; n = 28, p = 0.032) and lactate (r_s_ = −0.448; n = 28, p = 0.017) levels at 140 minutes.

### Sex differences

There were no differences in STAI-trait or PSS levels between sexes (Table 1). The baseline PPTs were significantly higher in men than in women on the hypertonic saline side (p = 0.023) but not on the isotonic saline side (p = 0.106). The PPT of the index finger did not differ between sexes. The PPTs assessed after microdialysis had decreased on the hypertonic side in men (p = 0.023) but not on the isotonic saline side (p = 0.076), whereas there were no significant changes in the women (p = 0.308 for the hypertonic saline side and p = 0.121 for the isotonic saline side) (Table 3). Pain intensity, pain duration and pain area evoked by the injections did not differ between sexes (Table 2 and Figure 3). Furthermore there were no differences in the levels of 5-HT, glutamate, lactate, pyruvate, glucose or glycerol between sexes at any time point (data not shown).

## Discussion

This study investigated if experimentally induced muscle pain by hypertonic saline injection into the masseter muscle lead to a release of muscle biomarkers, sampled using intramuscular microdialysis. The main findings were that 5-HT, glutamate and glycerol levels increased in response to the painful hypertonic saline injection. There were also significant correlations between pain intensity and the levels of 5-HT and glycerol. Interestingly, no sex differences were found in the release of biomarkers.

No previous study has investigated muscle 5-HT levels in response to hypertonic saline injection. However, one study reported that hypertonic saline injection into bicep muscles induced a release of glutamate [24], in accordance with the findings of the present study. Other studies reported no release of 5-HT or glutamate in the masseter muscle after experimental tooth clenching or acidic saline injection [40,41]. The difference between studies might be attributed to the differences in methodology. In addition, these two experimental pain models evoked lower levels of pain compared to hypertonic saline in the present study. This indicates that the hypertonic saline model that clinically can be regarded a valid experimental model for myalgia, also seem to be valid with respect to glutamate and 5-HT release. Although the hypertonic saline model, at least to some extent resembled an acute pain condition, one could argue that it would increase the levels of 5-HT and glutamate, such as in chronic pain due to its mechanism. Hypertonic saline has been shown to induce pain by activation of sodium channels but also by the release of glutamate [24]. Furthermore, another study points out that the pro-nociceptive effect could possibly be due to the release of 5-HT acting on 5-HT_3_ –receptors or even by direct activation of the 5-HT_3_ –receptors [31].

Several independent studies have shown increased levels of 5-HT and glutamate in clinical myalgia and positive correlations to muscle pain and tenderness [16,15,42,18,43,19]. In the present study there was a positive correlation between 5-HT and pain intensity, which was in line with previous studies [39,44] and strengthens the validity of 5-HT as an important biomarker in muscle pain. Other studies showed a positive correlation between glutamate and pain in patients with TMD and trapezius myalgia [19,16]. No such correlation was found in this study after injection of hypertonic saline, but there was a negative correlation on the control side between glutamate and pain level. However, this negative correlation was considered without significance since the pain evoked by isotonic saline was minimal and there were large variations in mediator levels.

Interestingly there was also an increased release of glycerol in response to the hypertonic saline injection in this study. Previous studies have suggested that glycerol may be a marker of cell membrane injury [45]. Loss of energy due to ischemia and/or mitochondrial dysfunction eventually leads to an influx of calcium and a decomposition of cell membranes, which liberates glycerol into the interstitial fluid [45]. Increased levels of glycerol may also serve as a marker of muscle damage after surgery [46]. On the other hand, glycerol levels correlated negatively to pain level, which may seem contradictory. However, since there is just limited knowledge about the relationship between glycerol and muscle pain, no certain conclusions can be drawn. Hence, further studies are needed to evaluate any possible relationships between pain and glycerol.

There was no release of lactate and pyruvate, which was in line with previous studies, inducing pain in the bicep muscles and masseter muscle with hypertonic saline and acidic saline, respectively [24,40]. Other studies reported increased levels of pyruvate and lactate in patients with chronic trapezius myalgia [19,43,47-49] and also in patients with fibromyalgia and chronic widespread pain [17,50]. In clinical conditions, higher levels of lactate and pyruvate may be due to changes in the lactate-pyruvate metabolism. However, experimental pain models might differ from clinical conditions and do not seem to influence muscle metabolism, perhaps because of their acute character. On the other hand, lactate correlated negatively to pain levels on the control side. As with glutamate, the significance of this finding is unknowns because of the low pain level evoked by isotonic saline. In line with previous studies of patients with chronic trapezius myalgia, there was no release of glucose [49].

The significant decrease in PPT on both sides after the microdialysis was not surprising since it has been described in a previous study [25]. This might be explained by the trauma caused by the needle and probe when inserting the catheter, sensitizing the muscle tissue. Although there are few studies showing reduced muscle PPTs after hypertonic saline injections, most studies report no effect on PPTs [51].

One might claim that the release of biomarkers could have been affected by the insertion of the microdialysis probe. However, previous studies have shown that lactate, pyruvate and 5-HT return to baseline levels within 1 hour after insertion of the microdialysis catheters [44,52]. Therefore, a stabilization period of 2 hours should have been sufficient. This is also a time frame commonly used for the trauma phase in other studies [18,19,17]. Another issue could be that we used topical anesthetics (EMLA cream) to anesthetize the skin before insertion of the microdialysis catheter, which possibly might influence the interstitial muscle levels of biomarkers. However, this is unlikely since a previous study has shown that EMLA cream applied for 30 minutes on the forearms did not penetrate deeper than 2.5 mm [53]. This is insufficient to affect the underlying masseter muscle, hence the local anesthesia most likely had no effect on the reported release of biomarkers.

Although RR was slightly higher at the beginning of the microdialysis, the magnitude in general was in line with previous studies using similar microdialysis probes [41,44]. This indicates that the calculated interstitial levels of the substances were comparable between studies. However, the RR was lower than in studies of other muscles where a much longer probe could be used. This was not surprising since it is well known that the RR, among other factors, depends on the size of the membrane area [18,19,17].

There were no changes in the blood flow after injections. This is in line with previous study [41] in which experimental pain was induced by repeated tooth clenching. However, it cannot be excluded that blood flow change might have occurred, but could not be detected, since the dialysates were sampled every 20th minute. Minute changes might therefore be undetectable.

In this study there were no sex differences regarding the release of biomarkers. A previous study from this research group where microdialysis was performed during an experimental pain setting with acidic saline showed significantly more samples with detectable levels of 5-HT in women than men [40]. However, as in the present study, the levels of 5-HT (and the other biomarkers) did not differ between sexes. One could argue that the sample size was too small to detect sex differences, which was a limitation of the study. Indeed, the study was primarily powered to detect changes in mediator levels after hypertonic saline for the group as a whole. However, tendencies were not even detected in sex differences related to the release of biomarkers, suggesting that this may be a true finding. Moreover, even if a much stronger pain was evoked by hypertonic saline compared to acidic saline, the VAS peak did not differ between men and women. This was in line with a previous study of hypertonic saline-induced masseter myalgia in a sample of similar size [34]. On the other hand, that study reported twice as large pain area in the women. In addition in this study women reported a larger pain area, although the difference between sexes was smaller and did not reach significance.

## Conclusion

The results of this study showed that a single injection of hypertonic saline in the human masseter muscle evoked muscle pain and an increased muscle release of 5-HT, glutamate and glycerol, but there were no sex differences. Since increased levels of 5-HT and glutamate have been reported in chronic myalgia, this strengthens the validity of the pain model.

## Abbreviations

TMD: Temporomandibular disorders; 5-HT: 5-hydroxytryptamine; serotonin; VAS: Visual analogue scale; PPT: Pressure pain threshold; RDC/TMD: Research Diagnostic Criteria for temporomandibular disorders; STAI-trait: State-Trait Anxiety Inventory; PSS: Perceived Stress Scale; RR: Relative recovery; NRS: Numeric rating scale.

## Competing interests

The authors declare that they have no competing interests.

## Authors’ contributions

SL responsible for data collection, contributed in the statistical analysis, and is the main author of the manuscript. NC contributed in the data collection, the statistical analysis and in writing of the manuscript in general. BG performed the chemical analysis, contributed to the statistical analysis and revising the article. BG contributed to the design of the study and revising the article. PS contributed to the design of the study and revising the article. TL contributed to the design of the study and revising the article. ME Responsible to the design of the study, contributed to the statistical analysis, and in writing of the manuscript in general. All authors read and approved the final manuscript.
